# Coping with COVID: Performance of China’s hierarchical medical system during the COVID-19 pandemic

**DOI:** 10.3389/fpubh.2023.1148847

**Published:** 2023-04-27

**Authors:** Yong Yang, Lieyu Huang, Hao Yan, Stephen Nicholas, Elizabeth Maitland, Qian Bai, Xuefeng Shi

**Affiliations:** ^1^Health Management Center, General Practice Medical Center, West China Hospital, Sichuan University, Chengdu, China; ^2^Medical Device Regulatory Research and Evaluation Center, West China Hospital, Sichuan University, Chengdu, China; ^3^School of Management, Beijing University of Chinese Medicine, Beijing, China; ^4^Office of Policy and Planning Research, Chinese Center for Disease Control and Prevention (China CDC), Beijing, China; ^5^Australian National Institute of Management and Commerce, Sydney, NSW, Australia; ^6^Guangdong Institute for International Strategies, Guangdong University of Foreign Studies, Guangzhou, China; ^7^School of Economics and School of Management, Tianjin Normal University, Tianjin, China; ^8^Newcastle Business School, University of Newcastle, Callaghan, NSW, Australia; ^9^University of Liverpool Management School, University of Liverpool, Liverpool, United Kingdom; ^10^State Key Laboratory of Quality Research in Chinese Medicine, Institute of Chinese Medical Sciences, University of Macau, Taipa, Macau, China; ^11^National Institute of Traditional Chinese Medicine Strategy and Development, Beijing University of Chinese Medicine, Beijing, China

**Keywords:** health service, COVID-19, public hospital, hierarchical medical system, healthcare-seeking behavior

## Abstract

**Objective:**

The COVID-19 pandemic has challenged the health system worldwide. This study aimed to assess how China’s hierarchical medical system (HMS) coped with COVID-19 in the short-and medium-term. We mainly measured the number and distribution of hospital visits and healthcare expenditure between primary and high-level hospitals during Beijing’s 2020–2021 pandemic relative to the 2017–2019 pre-COVID-19 benchmark period.

**Methods:**

Hospital operational data were extracted from Municipal Health Statistics Information Platform. The COVID-19 period in Beijing was divided into five phases, corresponding to different characteristics, from January 2020 to October 2021. The main outcome measures in this study include the percentage change in inpatient and outpatient emergency visits, and surgeries, and changing distribution of patients between different hospital levels across Beijing’s HMS. In addition, the corresponding health expenditure in each of the 5 phases of COVID-19 was also included.

**Results:**

In the outbreak phase of the pandemic, the total visits of Beijing hospitals declined dramatically, where outpatient visits fell 44.6%, inpatients visits fell 47.9%; emergency visits fell 35.6%, and surgery inpatients fell 44.5%. Correspondingly, health expenditures declined 30.5% for outpatients and 43.0% for inpatients. The primary hospitals absorbed a 9.51% higher proportion of outpatients than the pre-COVID-19 level in phase 1. In phase 4, the number of patients, including non-local outpatients reached pre-pandemic 2017–2019 benchmark levels. The proportion of outpatients in primary hospitals was only 1.74% above pre-COVID-19 levels in phases 4 and 5. Health expenditure for both outpatients and inpatients reached the baseline level in phase 3 and increased nearly 10% above pre-COVID-19 levels in phases 4 and 5.

**Conclusion:**

The HMS in Beijing coped with the COVID-19 pandemic in a relatively short time, the early stage of the pandemic reflected an enhanced role for primary hospitals in the HMS, but did not permanently change patient preferences for high-level hospitals. Relative to the pre-COVID-19 benchmark, the elevated hospital expenditure in phase 4 and phase 5 pointed to hospital over-treatment or patient excess treatment demand. We suggest improving the service capacity of primary hospitals and changing the preferences of patients through health education in the post-COVID-19 world.

## Introduction

Beginning with the first reported coronavirus disease-19 (COVID-19) in Wuhan, the COVID-19 pandemic has challenged the health system worldwide (1, 2). China’s zero COVID-19 tolerance strategy involves local and regional lockdowns, large-scale compulsory testing, reduced travel, social distancing measures, and changed hospital use. Hospitals played a crucial role in saving lives and curbing the spread of the virus. But hospitals were also hazardous places, where non-COVID-19 patients might catch COVID-19, a major reason patients avoided hospitals, frequently going without necessary preventative and active treatments (3). Besides a fall in demand for medical services, the supply of services also declined as hospitals reduced many normal medical services while prioritizing Covid-related and emergency services. How did China’s hierarchical medical system (HMS) cope with the COVID-19 pandemic? We assess the performance of Beijing’s hierarchical medical system during the COVID-19 pandemic between January 2020 and October 2021. We measure the number and distribution of primary and high-level hospital visits, emergency visits, surgeries, and medical expenditures during the pandemic relative to the pre-COVID-19 period. Second, we measure the speed, or time, that HMS took to return to pre-COVID-19 levels of hospital visits and expenditures. Our analysis informs researchers and policymakers both in China and other countries about a mega-city’s medical system responses to short-and medium-term challenges during the COVID-19 public health emergencies.

A unique, and complicating feature of China’s HMS is patients’ first preference for high-level hospitals, rather than lower-level primary hospitals, for medical treatment (4). Patients’ high-level hospital first preference means high-level hospitals are over-used by patients, when non-emergency and common medical conditions can be adequately treated at primary health facilities. Primary hospitals are not used as gatekeepers, which helps explains much of the low efficiency, poor preventative care, and inadequate treatment regimes in Chinese hospitals (5). To address the over-use of high-level hospitals, improve efficiency and reduce waste, two decades of reform have seen the Chinese government implement a hierarchical medical system (HMS). Primary care hospitals, including village clinics, township hospitals, and community health centers, provide preventive and primary care services. High-level hospitals, including tertiary hospitals and provincial hospitals, provide comprehensive treatments, complex care, and medical research and training (5). China’s HMS involves four key parts: primary treatment at the community level; disparate treatment for emergency and chronic diseases; two-way referral; and cooperation between different-level medical facilities (6).

But two decades of reform have not seen a re-distribution of patient use from high-level to primary hospitals. From 2010 to 2019, the proportion of outpatient care provided by primary hospitals fell from 63.9 to 54.1%, and inpatient primary care dropped from 29.3 to 16.9%, of all hospital treatments (7). High-level hospitals comprised just 3.5 percent of medical institutions, but accounted for 45 percent of all outpatient visits (8). One study reported that nearly half of the patients in Shanghai’s high-level hospitals suffered from common or frequently-occurring diseases that could be adequately treated at primary hospitals (9). Despite the government’s increased health system expenditures and 15 years of health system reforms (10, 11), shift of patient preferences towards primary hospital care has been largely unsuccessful.

Against this background of hospital use, how to improve the performance of the HMS has been a research hotspot (4, 12, 13). COVID-19 challenged China’s city hospitals, as well as hospitals and health systems around the world. The evaluation and comparison of hospitals and healthcare systems’ performance pre-and during COVID-19 have garnered the attention of numerous scholars and policymakers (14). For example, a study evaluated the performance of Portuguese public hospitals using a network data envelopment analysis model and found a consistent decrease in efficiency during the pandemic, followed by a recovery to levels exceeding those prior to the pandemic (15). Another study estimated 55 nations’ efficiency in the fight against the pandemic (16). Zhou et al. calculated the health service efficiency of primary healthcare institutions among 28 provinces in China before COVID-19 and compared compare the urban–rural differences (17). Banafsheh Sadeghi and colleagues evaluated COVID-19 pandemic preparedness and performance in 180 countries using the key outcome measure COVID-19 fatality. A systematic review investigated the impact of the COVID-19 pandemic on the utilization of healthcare services and reported that healthcare utilization decreased by about a third during the pandemic (18). However, no study was found concerning the performance of the HMS in China during the pandemic.

With a well-established HMS, Beijing city is considered one of the medical centers in China and can be a representative example city. Using monitoring data on the operation of 361 Beijing primary and high-level hospitals, we examined how Beijing’s HMS coped in the short and medium-term to five phases of the COVID-19 pandemic: phase 1 outbreak, phase 2 epidemic, phase 3 sporadic COVID-19, phase 4 vaccinations, and phase 5 post-epidemic. Each COVID-19 phase impacted people’s healthcare-seeking behavior and supply of hospital services (19–21). By measuring the distribution and changes in patient visits (including non-local patients), surgery cases, and medical expenditures across Beijing’s primary and high-level hospitals, we assessed how the HMS coped during COVID-19 in comparison with the pre-COVID-19 period and the speed of returning to pre-COVID-19 treatment levels.

## Methods

### Study design

All data were analyzed at an aggregate level and no individual participants were included. We extracted monthly data on the number of outpatients and inpatients, emergency and surgery patients, and expenditure data from the Beijing Municipal Health Statistics Information Platform database. Our data from January 2017 to October 2021 covered 361 public hospitals, comprising 206 primary health care facilities and 155 high-level hospitals. The COVID-19 period was 22 months long spanning January 2020 to October 2021, and the baseline data were 36 months comprising 2017, 2018, and 2019. A total of 20,938 hospital-months data were included in the analysis. Confirmed cases of COVID-19 were collected from daily reports on the Beijing Municipal Health Commission website and summed to calculate monthly data.

Starting with the Wuhan COVID-19 outbreak, containment strategies, such as lockdown, home isolation, mass testing and intensive surveillance, were implemented by the health authorities (22). Since then, zero-tolerance COVID-19 prevention and control strategies have become the norm in China. Based on the number of Beijing COVID-19 cases, we defined five COVID-19 phases: phase 1 outbreak (2020/01/01–04/30), phase 2 epidemic (2020/05/01–08/31), phase 3 sporadic COVID-19 (2020/09/01–12/31), phase 4 vaccination (2021/01/01–04/31) and phase 5 post-epidemic (2021/05/01–10/31) (22, 23). These phases provided delineated periods to evaluate how Beijing’s HMS coped with the COVID-19 pandemic.

### Performance measures

Table 1 lists the outcome measures and their interpretation, comprising the percentage change in inpatient and outpatient emergency visits and surgeries and the changing distribution of patients between primary and high-level hospitals across Beijing’s HMS. These measures provide a measure of the phased changes in hospital use. The percentage change indicators were calculated as:


$$\left(\frac{\mathrm{Indicators during the COVID}-19\;\mathrm{period}}{\mathrm{The baseline level}\;\left(2017\sim 2019\right)}-1\right)*100\%$$

Compared with the baseline pre-COVID-19 level (2017–2019), we expected a large absolute decline in the percentage change indicators, corresponding to the largest number of COVID-19 cases that occurred in phase 1 and phase 2 (24). We expected the decline in high-level hospital visits, especially in phases 1 and 2, to be significantly greater than that at primary hospitals. There were two forces at work: first, primary hospitals were viewed as posing a lower risk of catching COVID-19 than high-level hospitals and, second, some non-essential departments in high-level hospitals shut down at the beginning of the pandemic, forcing patients to primary hospitals (4, 25, 26). Non-local patients, or patients without Beijing household registration, we anticipated followed the same pattern as local patients, but suffered greater falls. As measures of complex medical problems, we expected that surgeries and emergencies also fell in high-level hospitals. We assessed Beijing’s HMS coping well during the 2020–2021 pandemic by its return to 2017–2019 benchmark levels; the speed, or timing, of returning to 2017–2019 benchmark levels; and whether patient preferences for treatments shifted from over-used high-level to under-used primary hospitals.

**Table 1:** Interpretation of the main performance indicators.

Indicators	Corresponding results	Comments	HMS performance
The number of outpatients and inpatients	Figure 2	Reflecting change of total number of outpatients and inpatients	Details below
Percentage change of emergency visits to high-level hospitals & percentage change of surgery inpatients at high-level hospitals	Figure 3	Mainly measure urgent or critical medical needs	Return to 2017–2019 benchmark levels of emergency visits and surgery inpatients timely treated
The proportion of surgery outpatients in primary hospitals	Figure 4	Partly measure common medical needs	Return to 2017–2019 benchmark levels Change: Primary hospitals treated more minor surgery patients than pre-COVID-19 period
Percentage change of outpatients in primary and high-level hospitals	Figure 5	Mainly measure patients’ preference in choosing hospitals	Return to 2017–2019 benchmark levels Change: Primary hospitals treated more patients with minor medical needs than pre-COVID-19 period
The proportion of outpatients in primary hospitals	Figure 6	Ditto	Return to 2017–2019 benchmark levels Change: High-level hospitals larger decline in outpatients than primary hospitals
Percentage change of non-local patients	Figure 7	Reflecting the preference of non-local patients	Return to 2017–2019 benchmark levels Change: Non-local patients fall in high-level hospitals
Outpatient and inpatient expenditure	Figure 8	Reflecting change of health expenditure corresponding to the HMS	Details below

Each shadow area denotes COVID-19 phase 1 to phase 5. The green and red shading correspond to the part of higher expenditure for baseline than pandemic era. The yellow shading corresponds to the part of higher expenditure for the pandemic era than baseline.

### Statistical analysis

Three-year 2017–2019 mean values were calculated to provide pre-COVID-19 baseline comparative data. Considering the aim of the current study, to assess the HMS by measuring the number and distribution of hospital visits and healthcare expenditure between primary and high-level hospitals before-and during COVID-19, descriptive analysis was mainly employed. The method and indicators were widely used in the literature (27–29). First, the monthly total number of outpatients and inpatients was calculated in different COVID-19 phases, compared with the 2017–2019 baseline. This allowed us to assess the speed that the HMS treatments during the COVID-19 phases took to return to pre-COVID-19 levels. Second, we evaluated the percentage changes of emergency outpatients and surgery inpatients in high-level hospitals to identify the influence of the COVID-19 on urgent medical needs during different COVID-19 phases. Third, the proportion of surgery outpatients in primary hospitals and the proportion of surgery outpatients in high-level hospitals were calculated to reflect the changes in patients’ healthcare-seeking preferences. Fourth, the proportion of outpatients in primary hospitals and percentage changes of outpatients in primary and high-level hospitals were calculated to reflect changing patient preferences between primary hospitals and high-level hospitals during the COVID-19 pandemic. Similarly, the percentage change of non-local patients was also assessed in this phase. Finally, we compared the outpatient and the inpatient expenditure in different COVID-19 phases with the baseline level, to identify whether and how much health expenditure under HMS was affected due to changes in patients’ healthcare-seeking preferences.

## Results

### COVID-19 pandemic in Beijing

**Figure 1:**
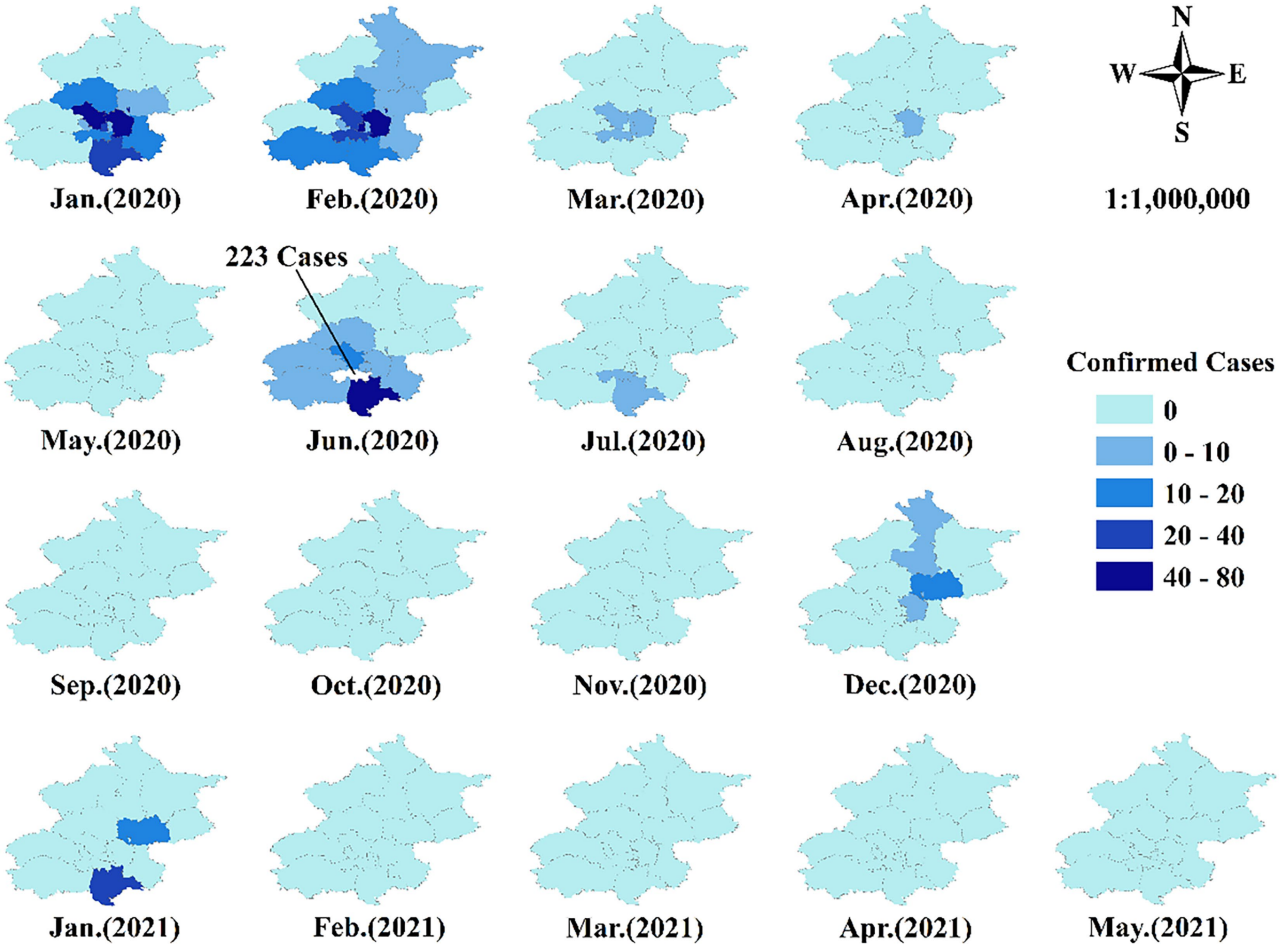
Confirmed local cases of COVID-19 from January 2020 to May 2021 in Beijing.

Figure 1 displays the monthly confirmed COVID-19 cases from January 2020 to May 2021 in Beijing. We did not include the non-Beijing, or imported cases, as these patients were treated in designated hospitals. As shown in Figure 1, January and February 2020 were the severest COVID-19 months with the largest number of confirmed cases widely spread across Beijing’s districts. All confirmed cases were discharged from hospitals in April 2020, ending the outbreak phase 1. In June 2020, COVID-19 emerged in the Xinfadi vegetable market district and spread to several surrounding districts, marking the epidemic stage, with this outbreak controlled in July 2020. The sporadic COVID-19 phase 3 in Figure 1 encompassed the subsequent months, with COVID-19 cases in December 2020 and January 2021. From February 2021, Figure 1 shows that there have been no confirmed new local cases for the 4 months to May 2021, during the vaccination phase 4. The phase 5 post-epidemic ran from June 2021.

### Change in the total number of outpatients and inpatients

**Figure 2:**
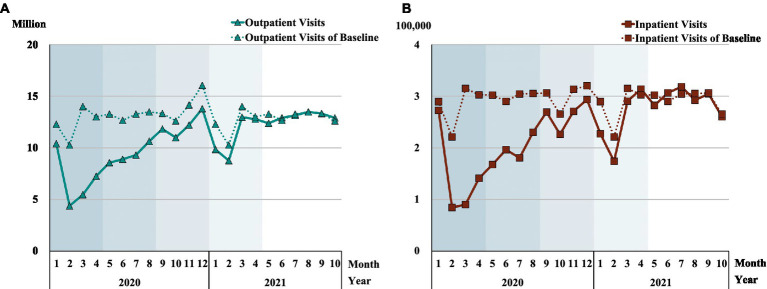
Number of 2020–2021 patients compared with benchmark pre-COVID-192017–2019 patients. Each shadow denotes COVID-19 phase 1 to phase 5. **(A)** demonstrates the change in outpatient visits, and **(B)** shows the change in inpatient visits.

Compared with the baseline data, Figure 2 shows that the total number of patient visits dramatically declined in phase 1 (outpatients decreased by 44.6% and inpatients decreased by 47.9%). The number of patients avoiding hospitals was significantly greater than any increase in COVID-19 patients. The plots of both outpatients and inpatient visits in Figure 2 display an upward trend in phase 2 with patients returning to hospitals, but the number of patients remaining below the pre-COVID-19 baseline until phase 4. There was a rapid return toward benchmark levels in phase 1 and phase 2. But the phase 3 gap to 2017–2019 levels was 13.1% for outpatients and 12% for inpatients, and remained 1.8% below pre-pandemic levels for outpatients in phase 4, although the number of phase 4 inpatients was 3.7% higher than the mean 2017–2019 benchmark. While the speed towards pre-COVID-19 levels was rapid in phase 1 and phase 2, the pre-COVID-19 levels were not attained before phase 4 for inpatients and phase 5 for outpatients (Supplementary Table 1).

### Change in the number of emergency outpatients and surgery inpatients in high-level hospitals

**Figure 3:**
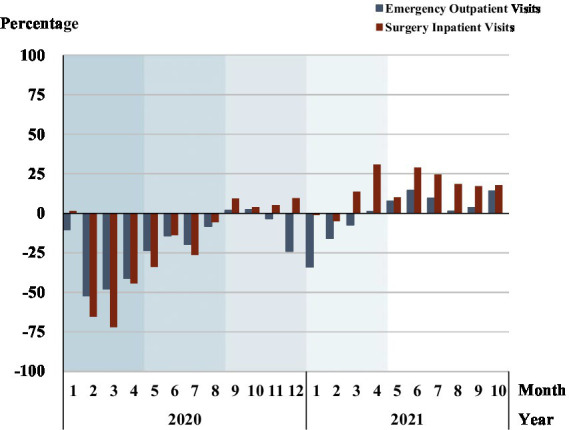
Change in number of high-level hospital inpatient surgeries and emergency outpatient visits. Each shadow area denotes COVID-19 phase 1 to phase 5.

The overall inpatient and outpatient visits in Figure 2 disguise the significant differences in hospital visits between high-level and primary hospitals. Figure 3 and Supplementary Table 2 depict the percentage change of emergency outpatients and surgery inpatients in high-level hospitals. There was a dramatic decline in both emergency outpatients (35.6%) and surgery inpatients (44.5%) relative to the 2017–2019 baseline. The largest drop occurred in February phase 1 among emergency outpatients (52.2%), and in March phase 1 for surgery inpatients (71.8%). In phase 2, both the emergency outpatients and surgery inpatients’ numbers narrowed relative to the baseline, with a large fluctuation in July as new COVID-19 cases re-emerged. The speedy return towards pre-pandemic benchmarks saw surgery inpatients reach their 2017–2019 baseline in phase 3. But the number of emergency outpatient visits declined in December phase 3 and January and March phase 4, not reaching their 2017–2019 benchmark until phase 5. In phase 5, the number of surgery inpatients was slightly lower than the benchmark in the first 2 months in 2021, but significantly higher in the subsequent 8 months, where inpatient surgeries were 25% above baseline by April 2021, probably reflecting catch-up treatments.

### Surgery outpatients in primary hospitals

**Figure 4:**
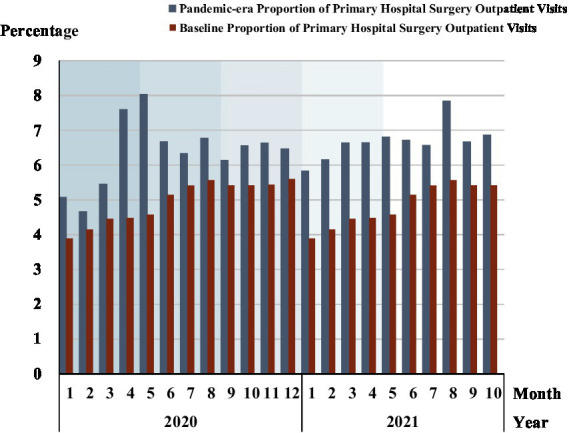
Proportion of surgery outpatients in primary hospitals. Each shadow area denotes COVID-19 phase 1 to phase 5.

Figure 4 and Supplementary Table 2 reveals the proportion of surgery outpatients in primary hospitals both in the COVID-19 and in the baseline period. Primary hospitals had a large overall increase in the proportion of surgery outpatients relative to the baseline during the pandemic period and peaked in phase 2. Primary hospitals coped with the pandemic by providing more surgery services for patients in the severest phase 1 and phase 2 of the pandemic. Although the percentage of primary hospital surgery outpatients declined in phase 3, it fluctuated around 1% above pre-COVID-19 levels, rising to 6.5% in phase 4. Though lower than in phase 4, the proportion of outpatient surgeries in primary hospitals in phase 5 was 1.6% higher than baseline on average.

### Performance of high-level and primary hospital outpatient treatment

**Figure 5:**
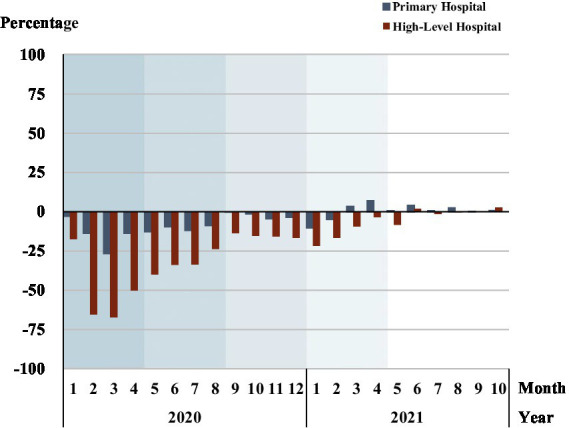
Percentage change of outpatients in primary and high-level hospitals. Each shadow area denotes COVID-19 phase 1 to phase 5.

Figure 5 displays the percentage change of outpatients in primary and high-level hospitals compared to the pre-COVID-19 baseline. In phase 1, outpatients in high-level hospitals displayed a sharper drop than that in primary hospitals. The percentage change of outpatients in primary hospitals remained below the baseline, but the margin closed rapidly in phase 1 and phase 2, and was mostly closed in phase 3, but not fully closed until mid-phase 4. In high-level hospitals, Figure 5 shows that the outpatient visit gap with pre-pandemic levels was not closed until phase 5, with phase 1 and phase 2 showing a speedy narrowing of the gap, which stalled in period 3 and period 4.

**Figure 6:**
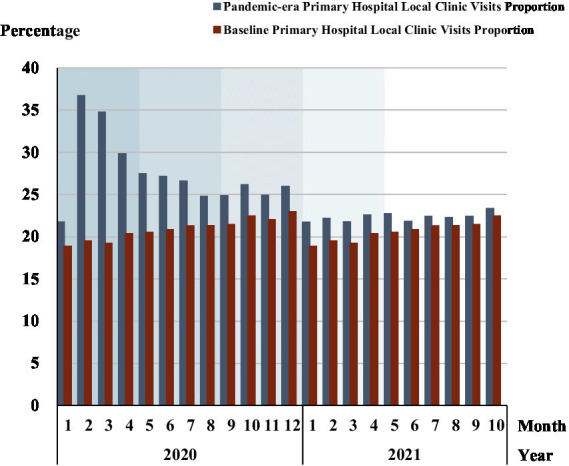
The proportion of outpatients in primary hospitals. Each shadow area denotes COVID-19 phase 1 to phase 5.

Figure 6 and Supplementary Table 3 show the proportion of outpatients in primary hospitals rose relative to outpatients in all the hospitals, reflecting a patient preference for primary hospitals. The percentage of primary hospital outpatients relative to all hospital outpatients peaked in February 2020 (36.7%), then fell until phase 5. Phase 1 had the largest increase in the proportion of outpatients in primary hospitals, 11.3% (30.7% vs. 19.5%). While in phase 5, the primary hospitals had only a 1% higher proportion of outpatients than the baseline level.

### Non-local patients in high-level hospitals in Beijing

**Figure 7:**
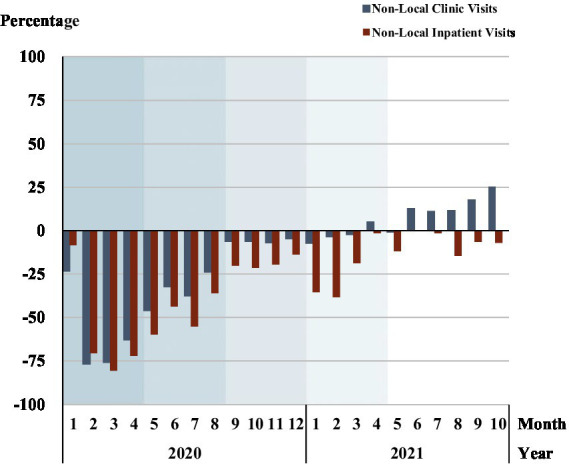
Percentage change in non-local patient visits in high-level hospitals. Each shadow area denotes COVID-19 phase 1 to phase 5.

Figure 7 and Supplementary Table 3 illustrate the percent changes in non-local patient clinic visits, including emergency visits and outpatient visits, and non-local inpatients in high-level hospitals in Beijing. Both non-local patient clinic visits and non-local inpatient visits dramatically declined in phase 1, especially in February and March 2020, where clinic visits decreased by 76.7% in February and inpatients visits decreased by 80.5% in March. Both clinic visits and inpatients from other provinces remained below the pre-COVID-19 baseline visits in phase 2, phase 3 and phase 4. In phase 3, the number of non-local inpatients was 5.90% less than the same stage of baseline and was 12.74% less than in phases 4 and 5. In phase 5, the percentage change in the number of non-local clinic visits was close to or exceeded the baseline from May 2021, with a percentage change about 25% greater than the baseline in October 2021.

### Comparative health expenditures

**Figure 8:**
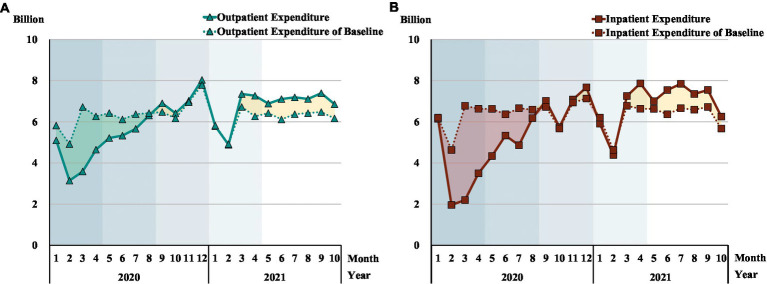
Comparison of the health expenditure pre and post COVID-19. Each shadow area denotes COVID-19 phase 1 to phase 5; **(A, B)** The green and red shading correspond to the part of higher expenditure for baseline than pandemic era; The yellow shading corresponds to the part of higher expenditure for the pandemic era than baseline.

Figure 8 displays health expenditures during the COVID-19 era and the baseline level. Compared with the pre-COVID-19 years, both the outpatient and inpatient health expenditure fell significantly in phase 1 and phase 2, with outpatient expenditure declining 30.5% and inpatient expenditure declining 43.1%. From the nadir in February 2020, both the outpatient and inpatient expenditures displayed an overall uptrend until the end of phase 3, tracking the baseline from November 2020, but with a lower number of patients. In terms of closing the gap with pre-COVID-19 levels, from April 2021 phase 4 outpatient and inpatient expenditures exceeded the baseline.

## Discussion

We assessed how Beijing’s HMS coped during the COVID-19 epidemic compared with the average 2017–2019 baseline level and also chartered the distribution of patient treatments and expenditures between primary and high-level hospitals relative to each other. In response to the pandemic, our data show that hospital visits fell, not attaining their pre-pandemic 2017–2019 benchmark until phase 4, although the pre-COVID-19 benchmark gap was narrowed in phase 1 and phase 2. Second, emergency services at high-level hospitals remained below the 2017–2019 benchmark until phase 2. Non-local patients fell at high-level hospitals through the entire pandemic period. In terms of patients with basic medical needs, Beijing’s HMS coped well during the early stage of the pandemic when patients accessed primary hospitals. However, the shift in the distribution of patients from over-used high-level to under-used primary hospitals during phases 1–3, was not sustained, with the pre-pandemic over-use of high-level hospitals restored by phase 4.

### COVID-19 impact on healthcare-seeking

The dramatic decline in outpatient and inpatient rates in the early phases reflected both supply and demand factors. On the demand side, patients avoided or delayed health treatments because of fear of catching COVID-19 and pandemic-induced restrictions, including a reduction of high-level hospital services (26). In a survey analyzing the factors that lead to delayed medical treatment for chronic disease patients during COVID-19 in China, the fear of catching COVID-19 in the hospital was ranked first (3). On the supply side, health service suspensions meant reduced visits, with the Beijing Municipal Health Commission in January 2020 canceling some non-emergency services, such as most stomatology services. Typical of high-level hospitals, the Beijing Hospital of Integrated Traditional Chinese and Western Medicine suspended services across 14 departments in February 2020, including sub-health management and ophthalmology. Current studies highlighted that the cessation of certain health services may lead to a decline in patients’ quality of life. Additionally, the healthcare system may incur higher costs in the future to regain the loss of benefits from previous therapies due to their discontinuation (30, 31). Thus, to reduce the unessential visits to hospitals and insure basic health services, the government implemented a series of policies including prompting pharmacy delivery services and telemedicine. Furthermore, the doctors were allowed to reasonably prescribe up to 12-week drugs for patients with chronic illnesses, such as hypertension, diabetes, and chronic obstructive pulmonary diseases (32).

Other studies have also revealed significant reductions in hospital visits during the early stage of the COVID-19 pandemic. US studies reported decreased emergency department (ED) visits ranging from 41.5 to 63.5% during the early pandemic period (33). In the 10 weeks following the US COVID-19 outbreak, ED visits declined by 23% for myocardial infarction, 20% for stroke, and 10% for the hyperglycemic crisis, compared with the preceding 10-week period (20, 34–36). In a tertiary referral center in Boston, 35.1% of hospitalizations decreased in the first 6 weeks compared with the same period in 2019 (37). Importantly, COVID-19 has posed a significant influence on patients’ healthcare-seeking behavior worldwide. Studies conducted in India (38), Lithuania (39), and Australia (40) identified delayed healthcare seeking from patients which was similar to our findings. Another study conducted in China also reported that the COVID-19 epidemic has greatly affected the behavior of tuberculosis patients seeking medical care, with some of them delaying or giving up healthcare seeking (41).

Similar to our results, patients were also observed returning to hospitals gradually, fluctuating with the change in the COVID-19 situation (42, 43). The recovery of hospital visits was rapid in phase 1-phase 3, but did not close the gap with pre-COVID-19 levels. Unlike many other countries, Beijing’s zero-tolerance strict prevention and control measures saw the COVID-19 outbreak brought rapidly under control. As the emergency response level was lowered, hospitals re-opened and suspended departments gradually provided services. As residents were vaccinated for COVID-19 during the phase 3 vaccination period, both outpatient and hospitalization rates in primary and high-level hospitals reached, or exceeded, their pre-COVID-19 baseline levels in phase 4 and phase 5.

### Rise and fall of visits to primary hospitals

During the early phases of COVID-19, the HMS in Beijing realigned patients to suitable medical resources. The proportion of surgery outpatient visits and common outpatient visits rose in primary hospitals relative to high-level hospitals. A 9.5% increase in outpatients in primary hospitals and a 59.4% decrease in non-local outpatients in high-level hospitals reversed a decade of increased use of high-level hospitals. This realignment of patients reflected a correction to the over-use of high-level hospitals, reflecting one chief aim of the HMS (6). In this respect, the pandemic brought a change in patient preferences for hospital treatment.

The realignment of patients towards high-level hospitals was managed through hospital appointments and a community referral system (44). Older patients and those suffering from chronic illnesses were strongly encouraged to receive primary diagnosis and treatment in primary healthcare facilities. Not only were patients with common or minor medical needs advised to visit primary hospitals, but patients were turned away from high-level to primary hospitals (45). Second, high-level hospitals equipped with a fever department meant patients turned towards primary hospitals due to the increased risk of catching COVID-19 in high-level hospitals. Third, telemedicine and pharmacies as alternatives to hospital visits quickly developed. Supported by the government, telemedicine became an important access point for health care during COVID-19 worldwide, mainly using telephone, video calls, and web servers to “visit” a doctor (19, 46, 47), which especially replaced high-level hospital visits.

The realignment of patient preferences for primary hospitals was not permanent. Patient preferences for high-level over primary hospitals were reversed by phase 3, and during phase 4 and phase 5 of the pandemic over-use (under-use) of high-level (primary) hospitals was re-established as a key feature of Beijing’s HMS. Our data on how Beijing’s HMS coped with the pandemic suggest that the task of reforming the use of China’s HMS is daunting. The greatest health crisis in 100 years failed to change patient hospital preferences in China. The task of changing patient preferences will require long-term significant new resources and targeted measures. Leveling up primary versus high-level hospital quality will be a long-term financial task requiring many years. Specifically, the quality of primary health care should be improved, with higher trained and qualified general practitioners and technology innovations, including telemedicine and effective management mechanisms needed by primary hospitals (48). These resource reallocations should reduce competition between primary and high-level hospitals and reduce the attraction of high-level Beijing hospitals to patients from other cities and regions (31). A more radical approach would involve discouraging patients from visiting high-level hospitals before using primary hospitals as gatekeepers. This could be incentivized through charging substantial differential fees for visiting high-level versus primary hospitals. Alternatively, high-level hospitals could require patients to receive a referral from a primary hospital. Only patient experiences with quality primary hospital healthcare will change patient preferences for primary hospitals. Other supporting measures will be required. High-level hospital appointment policy needs to discourage treatment for minor health issues more appropriately managed at primary hospitals. Targeted information campaigns are required to educate patients on primary hospital gatekeeping functions.

### Rise of health expenditures

Health expenditure was below the baseline in the first two COVID-19 phases, but reached pre-COVID-19 levels by phase 3. By mid-phase 4 (March 2021), both the outpatient and the inpatient health expenditure was higher than the 2017–2019 baseline, but without any growth in patient numbers. Compared to 2017–2019, the surging health expenditures suggest more expensive treatments for a stable number of patients. This might reflect over-servicing or patient over-demand for services or some combination of both (49). The government should strengthen supervision on over-treatment, especially unnecessary surgeries and over-diagnosing procedures to avoid both hospital waste and elevated out-of-pocket expenses for patients (50). While the phase 1 and phase 2 expenditure data reflects HMS coping with the pandemic, the rising expenditures surpassing the 2017–2019 baseline, points to a predicament of the HMS to manage healthcare costs, especially in the post-pandemic world.

### Strengths and limitations of the study

This is the first study of patient use and healthcare expenses during different phases (outbreak, epidemic, vaccination, sporadic COVID-19, and post-epidemic) in China’s pandemic. First, our data are hospital-level monitoring data, which means patients’ individual healthcare-seeking behavior could not be assessed. Future studies should assess patients’ healthcare-seeking behavior from the perspective of different disease groups using individual data. More finely differentiated patient data, such as critical versus common illnesses and treatment of children aged 0–4 would provide new insights. Second, expenditure data on private hospitals and pharmacies were not included, which means our study may not reflect the whole health system’s health expenditure. Since private hospitals and pharmacy were not included, health expenditure above baseline in phase 4 and phase 5 suggests our data under-estimated total healthcare expenditures. Third, phase 5 ended in October 2021 to avoid the confounding effects of the Beijing Olympic Winter Games, which meant no new pandemic wave was identified. Fourth, the Beijing results are likely to be representative of large city HMS, they may not reflect smaller cities.

## Conclusion

Beijing’s hospital system faced a large fall in hospital visits, emergency treatments and surgeries during the first phases of the COVID-19 pandemic. Beijing’s HMS only reach pre-pandemic 2017–2019 levels of treatments in the last phases (phase 4 and phase 5) of the pandemic. In the early pandemic phases, primary hospitals played an important role in guaranteeing healthcare needs as patients substituted primary hospital treatment for high-level hospitals. This redistribution of patients reflected a better allocation of patient healthcare use, as patients moved away from over-used high-level hospitals towards primary care. By the sporadic COVID-19 phase 3, inpatients and outpatient visits, emergency visits and surgeries approached, but only reached in phase 4 and phase 5, the 2017–2019 benchmark level. While the gap with benchmark levels was quickly narrowed, only in phase 4 or phase 5 was the gap closed.

We identified two important further findings. Medical expenditures in phase 4 and phase 5 may point to over-servicing by hospitals and over-demand healthcare by patients. Second, the pandemic did not permanently change patients’ preferences for high-level over primary hospitals. The failure to permanently change patient healthcare preferences means that the HMS faces the same challenges post-pandemic as in the pre-COVID-19 period. To strengthen the HMS in the post-pandemic world, we suggest the policy-makers focus on the following aspects: (1) Improving the service capacity of the primary hospitals in terms of the talent pool, medical technology, and medical equipment. (2) Enhancing health education for patients and guiding patients with chronic and minor illnesses to seek medical care at primary hospitals. More importantly, to change patients’ preference for high-level hospitals. (3) Strengthen the supervision of hospital and physician behavior to avoid excessive medical treatment after the epidemic.

## Data availability statement

The original contributions presented in the study are included in the article/Supplementary material, further inquiries can be directed to the corresponding author.

## Author contributions

XS, YY, and LH designed the study. YY and LH prepared the study data and performed the statistical analysis, which was quality checked by HY. All authors contributed to the interpretation of the findings. SN, EM, and QB wrote the original draft. All authors contributed to the article and approved the submitted version.

## Funding

This study was supported by key disciplines of the science of public management at the Beijing University of Chinese Medicine (No grant number); the Beijing Administration of Traditional Chinese Medicine (2040071520131).

## Conflict of interest

The authors declare that the research was conducted in the absence of any commercial or financial relationships that could be construed as a potential conflict of interest.

## Publisher’s note

All claims expressed in this article are solely those of the authors and do not necessarily represent those of their affiliated organizations, or those of the publisher, the editors and the reviewers. Any product that may be evaluated in this article, or claim that may be made by its manufacturer, is not guaranteed or endorsed by the publisher.

## References

[1] Cucinotta D Vanelli M. WHO declares COVID-19 a pandemic. Acta Biomed. (2020) 91:157–60. doi: 10.23750/abm.v91i1.9397, PMID: 32191675 PMC7569573

[2] Wu JT Leung K Leung GM. Nowcasting and forecasting the potential domestic and international spread of the 2019-nCoV outbreak originating in Wuhan, China: a modelling study. Lancet (London, England). (2020) 395:689–97. doi: 10.1016/s0140-6736(20)30260-9, PMID: 32014114 PMC7159271

[3] Ziyu W Xiyu H Yanhua H Hanwen G Yuan L Kaisheng F. Analysis of delayed medical treatment for chronic diseases patients during COVID-19 epidemic. Chin Hosp Manag. (2022) 42:43–7.

[4] Zhou Z Zhao Y Shen C Lai S Nawaz R Gao J. Evaluating the effect of hierarchical medical system on health seeking behavior: a difference-in-differences analysis in China. Soc Sci Med. (2021) 268:113372. doi: 10.1016/j.socscimed.2020.113372, PMID: 32979776

[5] Li X Lu J Hu S Cheng KK De Maeseneer J Meng Q. The primary health-care system in China. Lancet (London, England). (2017) 390:2584–94. doi: 10.1016/s0140-6736(17)33109-4 29231837

[6] Shen M He W Li L. Incentives to use primary care and their impact on healthcare utilization: evidence using a public health insurance dataset in China. Soc Sci Med. (2020) 255:112981. doi: 10.1016/j.socscimed.2020.112981, PMID: 32315873

[7] China NHCotPsRo. National Report on the Services, Quality and Safety in Medical Care System. China: Science and Technology Literature Press (2020) ISBN: 9787518970001.

[8] Li J Zhao N Zhang H Yang H Yang J. Patients' willingness of first visit in primary medical institutions and policy implications: a National Cross-Sectional Survey in China. Front Public Health. (2022) 10:842950–10. doi: 10.3389/fpubh.2022.842950, PMID: 35433566 PMC9010779

[9] Zuda L Xiaomei T Shu C Peiran C. Study on patient centered behavior of seeking medical treatment in different places-taking Shanghai as an example. China Health Insurance. (2020) 10:62–4. doi: 10.19546/j.issn.1674-3830.2020.10.014

[10] Fu W Zhao S Zhang Y Chai P Goss J. Research in health policy making in China: out-of-pocket payments in healthy China 2030. BMJ (Clinical Res Ed). (2018) 360:k234. doi: 10.1136/bmj.k234, PMID: 29437565 PMC5797981

[11] Han Y Yin W Wang A Li L Cheng C Chen Z. Study on the implementation difficulties and countermeasures of the hierarchical medical system based on the meter-horn model. Chinese journal of. Hosp Adm. (2019) 35:441–6. doi: 10.3760/j.issn.1000-6672.2019.06.002

[12] Li X Xu H Du F Zhu B Xie P Wang H. Does increasing physician volume in primary healthcare facilities under the hierarchical medical system help reduce hospital service utilisation in China? A fixed-effects analysis using province-level panel data. BMJ Open. (2023) 13:e066375. doi: 10.1136/bmjopen-2022-066375, PMID: 36822814 PMC9950906

[13] Sun J Yin M. The dilemma and countermeasure of the hierarchical diagnosis and treatment in China. Chin Med Ethics. (2018):236–40.

[14] Rathnayake D Clarke M Jayasinghe VI. Health system performance and health system preparedness for the post-pandemic impact of COVID-19: A review. (2021) 14:250–54. doi: 10.1080/20479700.2020.1836732

[15] Nunes AM Ferreira DFC. Evaluating Portuguese public hospitals performance: Any difference before and during COVID-19? Sustainability. (2023) 15:294. doi: 10.3390/su15010294

[16] Pereira MA Dinis DC Ferreira DC Figueira JR Marques RC. A network data envelopment analysis to estimate nations’ efficiency in the fight against SARS-CoV-2. Expert Syst Appl. (2022) 210:118362. doi: 10.1016/j.eswa.2022.118362, PMID: 35958804 PMC9355747

[17] Zhou J Peng R Chang Y Liu Z Gao S Zhao C. Analyzing the efficiency of Chinese primary healthcare institutions using the Malmquist-DEA approach: evidence from urban and rural areas. Front Publ Health. (2023) 11:1073552. doi: 10.3389/fpubh.2023.1073552, PMID: 36817900 PMC9931751

[18] Moynihan R Sanders S Michaleff ZA Scott AM Clark J To EJ. Impact of COVID-19 pandemic on utilisation of healthcare services: a systematic review. BMJ Open. (2021) 11:e045343. doi: 10.1136/bmjopen-2020-045343, PMID: 33727273 PMC7969768

[19] Baum A Kaboli PJ Schwartz MD. Reduced in-person and increased Telehealth outpatient visits during the COVID-19 pandemic. Ann Intern Med. (2021) 174:129–31. doi: 10.7326/m20-3026, PMID: 32776780 PMC7429994

[20] Jeffery MM D'Onofrio G Paek H Platts-Mills TF Soares WE 3rd Hoppe JA. Trends in emergency department visits and hospital admissions in health care systems in 5 states in the first months of the COVID-19 pandemic in the US. JAMA Intern Med. (2020) 180:1328–33. doi: 10.1001/jamainternmed.2020.3288, PMID: 32744612 PMC7400214

[21] Koonin LM Hoots B Tsang CA Leroy Z Farris K Jolly T. Trends in the use of Telehealth during the emergence of the COVID-19 pandemic-United States, January-march 2020. MMWR Morb Mortal Wkly Rep. (2020) 69:1595–9. doi: 10.15585/mmwr.mm6943a3, PMID: 33119561 PMC7641006

[22] Office TSCI. China's action against the COVID-19 epidemic. (2020). 5.7. (cited 2021 5.7). Available from: https://baijiahao.baidu.com/s?id=1668803769182611168&wfr=spider&for=pc.

[23] Government IOoBMPs. The emergency response level of Beijing's public health emergencies was adjusted to level three. (2020). 5.10. (cited 2021 6.16). Available from: http://www.beijing.gov.cn/ywdt/gzdt/202007/t20200719_1951751.html.

[24] Birkmeyer JD Barnato A Birkmeyer N Bessler R Skinner J. The impact of The COVID-19 pandemic on hospital admissions in The United States. Health Aff (Millwood). (2020) 39:2010–7. doi: 10.1377/hlthaff.2020.00980, PMID: 32970495 PMC7769002

[25] Hu H Wang R Li H Han S Shen P Lin H. Effectiveness of hierarchical medical system policy: an interrupted time series analysis of a pilot scheme in China. Health Policy Plan. (2023). doi: 10.1093/heapol/czad018 PMC10190962 36905394

[26] Czeisler M Marynak K Clarke KEN Salah Z Shakya I Thierry JM. Delay or avoidance of medical care because of COVID-19-related concerns-United States, June 2020. MMWR Morb Mortal Wkly Rep. (2020) 69:1250–7. doi: 10.15585/mmwr.mm6936a4, PMID: 32915166 PMC7499838

[27] Liu S Lin J He Y Xu J. The Service capability of primary health institutions under the hierarchical medical system. Healthcare (Basel, Switzerland). (2022) 10. doi: 10.3390/healthcare10020335, PMID: 35206949 PMC8872352

[28] Chen Q Haichao L. Evaluation on effect of comprehensive reform of separating drug sales from medical Services in Beijing: based on perspective of efficiency and accessibility. Hosp Admin J Chin PLA. (2020) 27:559–63. doi: 10.16770/J.cnki.1008-9985.2020.06.018

[29] Quanyu Z Yujie Y Xing L Haichao L. A study on evaluation of the efficiency and accessibility in the comprehensive reform of linkage between medical services and medical supplies in Beijing. Chin J Health Policy. (2021) 14:24–8. doi: 10.3969/j.issn.1674-2982.2021.01.004

[30] De Donno A Acella A Angrisani C Gubinelli G Musci G Gravili G. Suspension of Care for Patients with Spasticity during COVID-19 pandemic: ethical and medico-legal point of view starting from an Italian study. Front Med. (2021) 8:754456. doi: 10.3389/fmed.2021.754456, PMID: 34917632 PMC8669589

[31] Xiao H Dai X Wagenaar BH Liu F Augusto O Guo Y. The impact of the COVID-19 pandemic on health services utilization in China: time-series analyses for 2016-2020. Lancet Regional Health Western Pacific. (2021) 9:100122. doi: 10.1016/j.lanwpc.2021.100122, PMID: 34327438 PMC8315657

[32] Kang Y Shang W Liu Y Liu M. Policies implemented in Beijing for guaranteeing healthcare for community-dwelling patients withNoncommunicable diseases during the COVID-19 pandemic. Chin Gen Pract. (2022) 25:1172–6. doi: 10.12114/j.issn.1007-9572.2022.0091

[33] Ghaderi H Stowell JR Akhter M Norquist C Pugsley P Subbian V. Impact of COVID-19 pandemic on emergency department visits: a regional case study of informatics challenges and opportunities. AMIA Ann Symp Proceed AMIA Symp. (2021) 2021:496–505. PMC8861727 35308996

[34] Lange SJ Ritchey MD Goodman AB Dias T Twentyman E Fuld J. Potential indirect effects of the COVID-19 pandemic on use of emergency departments for acute life-threatening conditions-United States, January-may 2020. MMWR Morb Mortal Wkly Rep. (2020) 69:795–800. doi: 10.15585/mmwr.mm6925e2, PMID: 32584802 PMC7316316

[35] Hartnett KP Kite-Powell A DeVies J Coletta MA Boehmer TK Adjemian J. Impact of the COVID-19 pandemic on emergency department visits-United States, January 1, 2019-may 30, 2020. MMWR Morb Mortal Wkly Rep. (2020) 69:699–704. doi: 10.15585/mmwr.mm6923e1, PMID: 32525856 PMC7315789

[36] Walker LE Heaton HA Monroe RJ Reichard RR Kendall M Mullan AF. Impact of the SARS-CoV-2 pandemic on emergency department presentations in an integrated health system. Mayo Clin Proc. (2020) 95:2395–407. doi: 10.1016/j.mayocp.2020.09.019, PMID: 33153630 PMC7501771

[37] Anderson TS Stevens JP Pinheiro A Li S Herzig SJ. Hospitalizations for emergent medical, surgical, and obstetric conditions in Boston during the COVID-19 pandemic. J Gen Intern Med. (2020) 35:3129–32. doi: 10.1007/s11606-020-06027-2, PMID: 32700221 PMC7375703

[38] Goyal M Singh P Singh K Shekhar S Agrawal N Misra S. The effect of the COVID-19 pandemic on maternal health due to delay in seeking health care: experience from a tertiary center. Int J Gynaecol Obstet. (2021) 152:231–5. doi: 10.1002/ijgo.13457, PMID: 33128794 PMC9087665

[39] Aldujeli A Hamadeh A Briedis K Tecson KM Rutland J Krivickas Z. Delays in presentation in patients with acute myocardial infarction during the COVID-19 pandemic. Cardiol Res. (2020) 11:386–91. doi: 10.14740/cr1175, PMID: 33224384 PMC7666599

[40] Podubinski T Townsin L Thompson SC Tynan A Argus G. Experience of healthcare access in Australia during the first year of the COVID-19 pandemic. Int J Environ Res Public Health. (2021) 18:10687. doi: 10.3390/ijerph182010687 34682432 PMC8535411

[41] Yinyin X Fei H Hui C Ni W Xin D Wei C. The impact of COVID-19 on tuberculosis patients’ behavior of seeking medical care — China, 2020. China CDC Weekly. (2021) 3:553–6. doi: 10.46234/ccdcw2021.143, PMID: 34594934 PMC8392940

[42] Solomon MD Nguyen-Huynh M Leong TK Alexander J Rana JS Klingman J. Changes in patterns of hospital visits for acute myocardial infarction or ischemic stroke during COVID-19 surges. JAMA. (2021) 326:82–4. doi: 10.1001/jama.2021.8414, PMID: 34076670 PMC8173470

[43] Leeb RT Bitsko RH Radhakrishnan L Martinez P Njai R Holland KM. Mental health-related emergency department visits among children aged <18 years during the COVID-19 pandemic-United States, January 1-October 17, 2020. MMWR Morb Mortal Wkly Rep. (2020) 69:1675–80. doi: 10.15585/mmwr.mm6945a3, PMID: 33180751 PMC7660659

[44] Daily B. Secondary and tertiary hospitals in Beijing have all required appointment for non-emergency visits. Liu M, editor. (2020). (cited 2021 6.13). Available from: http://www.gov.cn/xinwen/2020-02/27/content_5483766.htm.

[45] Gan WH Lim JW Koh D. Preventing intra-hospital infection and transmission of coronavirus disease 2019 in health-care workers. Saf Health Work. (2020) 11:241–3. doi: 10.1016/j.shaw.2020.03.001, PMID: 32292622 PMC7102575

[46] Lam K Lu AD Shi Y Covinsky KE. Assessing telemedicine Unreadiness among older adults in the United States during the COVID-19 pandemic. JAMA Intern Med. (2020) 180:1389–91. doi: 10.1001/jamainternmed.2020.2671, PMID: 32744593 PMC7400189

[47] Patterson V. Neurological telemedicine in the Covid-19 era. Nat Rev Neurol. (2021) 17:73–4. doi: 10.1038/s41582-020-00438-9, PMID: 33257883 PMC7703718

[48] The L. A tiered health-care delivery system for China. Lancet (London, England). (2019) 393:1178. doi: 10.1016/s0140-6736(19)30730-5 30910288

[49] Berwick DM Hackbarth AD. Eliminating waste in US health care. JAMA. (2012) 307:1513–6. doi: 10.1001/jama.2012.362 22419800

[50] Morgan DJ Dhruva SS Coon ER Wright SM Korenstein D. 2019 update on medical overuse: a review. JAMA Intern Med. (2019) 179:1568–74. doi: 10.1001/jamainternmed.2019.3842, PMID: 31498374 PMC8608222

