# Supplementary material for: Coping with COVID: Performance of China’s hierarchical medical system during the COVID-19 pandemic
Source: Front Public Health. 2023 Apr 27;11:1148847. doi: 10.3389/fpubh.2023.1148847 (PMC10173579; doi:10.3389/fpubh.2023.1148847)
Supplement: Supplementary file 1 [file Table_1.DOCX]

## Supplementary Table 1 Outpatient/inpatient visits and outpatient/inpatient expenditure before- and during COVID-19

| **Phase** | **Month** | **Outpatient Visits (Million)** | | **Inpatient Visits (Hundred thousand)** | | **Outpatient Expenditure (RMB, Billion)** | | **Inpatient Expenditure (RMB, Billion)** | |
| --- | --- | --- | --- | --- | --- | --- | --- | --- | --- |
|  |  | **Before COVID-19** | **COVID-19 era (Percentage change, %)** | **Before COVID-19** | **COVID-19 era (Percentage change, %)** | **Before COVID-19** | **COVID-19 era (Percentage change, %)** | **Before COVID-19** | **COVID-19 era (Percentage change, %)** |
| Phase 1 | 1 | 12.29 | 10.41(-15.31) | 2.89 | 2.73(-5.80) | 5.82 | 5.10(-12.42) | 6.20 | 6.15(-0.93) |
|  | 2 | 10.27 | 4.38(-57.39) | 2.21 | 0.84(-61.81) | 4.92 | 3.14(-36.08) | 4.63 | 1.96(-57.62) |
|  | 3 | 14.01 | 5.46(-61.05) | 3.15 | 0.90(-71.40) | 6.72 | 3.59(-46.62) | 6.78 | 2.20(-67.52) |
|  | 4 | 13.00 | 7.26(-44.20) | 3.02 | 1.41(-53.35) | 6.27 | 4.65(-25.83) | 6.63 | 3.50(-47.2) |
|  | Subtotal | **12.39** | **6.87(-44.55)** | **2.82** | **1.47(-47.87)** | **5.93** | **4.12(-30.52)** | **6.06** | **3.45(****-43.07)** |
| Phase 2 | 5 | 13.28 | 8.56(-35.53) | 3.02 | 1.67(-44.61) | 6.43 | 5.21(-18.85) | 6.63 | 4.34(-34.57) |
|  | 6 | 12.66 | 8.89(-29.76) | 2.90 | 1.96(-32.26) | 6.12 | 5.33(-12.9) | 6.36 | 5.33(-16.18) |
|  | 7 | 13.27 | 9.29(-29.98) | 3.04 | 1.81(-40.63) | 6.37 | 5.66(-11.15) | 6.66 | 4.86(-26.99) |
|  | 8 | 13.48 | 10.63(-21.09) | 3.05 | 2.30(-24.62) | 6.42 | 6.30(-1.86) | 6.59 | 6.18(-6.17) |
|  | Subtotal | **13.17** | **9.35(-29.01)** | **3.00** | **1.94(****-35.33)** | **6.33** | **5.63(-11.06)** | **6.56** | **5.18(****-21.04)** |
| Phase 3 | 9 | 13.33 | 11.82(-11.31) | 3.06 | 2.69(-12.08) | 6.48 | 6.90(6.62) | 6.72 | 7.02(4.38) |
|  | 10 | 12.61 | 10.99(-12.82) | 2.65 | 2.26(-14.78) | 6.17 | 6.41(4.01) | 5.67 | 5.74(1.22) |
|  | 11 | 14.15 | 12.20(-13.78) | 3.13 | 2.71(-13.61) | 6.95 | 7.02(1.01) | 6.93 | 7.08(2.18) |
|  | 12 | 16.06 | 13.80(-14.09) | 3.20 | 2.94(-8.32) | 7.79 | 8.04(3.13) | 7.13 | 7.67(7.62) |
|  | Subtotal | **14.04** | **12.20(-13.11)** | **3.01** | **2.65(-****11.96)** | **6.85** | **7.10(3.65)** | **6.61** | **6.88(4.08)** |
| 2021  Phase 4&5 | 1 | 12.29 | 9.84(-19.89) | 2.89 | 2.27(-21.59) | 5.82 | 5.79(-0.56) | 6.20 | 5.91(-4.78) |
|  | 2 | 10.27 | 8.76(-14.71) | 2.21 | 1.74(-21.32) | 4.92 | 4.88(-0.71) | 4.63 | 4.39(-5.23) |
|  | 3 | 14.01 | 12.97(-7.42) | 3.15 | 2.90(-7.84) | 6.72 | 7.35(9.38) | 6.78 | 7.25(7.00) |
|  | 4 | 13.00 | 12.77(-1.79) | 3.02 | 3.14(3.73) | 6.27 | 7.26(15.92) | 6.63 | 7.86(18.57) |
|  | 5 | 13.28 | 12.37(-6.82) | 3.02 | 2.82(-6.66) | 6.43 | 6.88(7.13) | 6.63 | 7.00(5.67) |
|  | 6 | 12.66 | 12.92(2.00) | 2.90 | 3.06(5.7) | 6.12 | 7.11(16.18) | 6.36 | 7.54(18.5) |
|  | 7 | 13.27 | 13.14(-1.03) | 3.04 | 3.18(4.62) | 6.37 | 7.19(12.87) | 6.66 | 7.85(17.8) |
|  | 8 | 13.48 | 13.49(0.07) | 3.05 | 2.92(-4.21) | 6.42 | 7.11(10.7) | 6.59 | 7.35(11.57) |
|  | 9 | 13.33 | 13.32(-0.09) | 3.06 | 3.05(-0.52) | 6.48 | 7.39(14.16) | 6.72 | 7.55(12.26) |
|  | 10 | 12.61 | 12.9(2.3) | 2.65 | 2.6(-1.94) | 6.17 | 6.85(11.15) | 5.67 | 6.25(10.22) |
|  | **Subtotal** | **12.82** | **12.25(-4.46)** | **2.90** | **2.77(-4.55)** | **6.17** | **6.78(9.92)** | **6.29** | **6.89(9.66)** |

## Supplementary Table 2 Emergency Outpatients, surgery inpatients in High-level hospitals and Proportion of surgery outpatients in primary hospitals befor- and during COVID-19

| Phase | Month | **Emergency Outpatients in High-level hospitals (Hundred thousand)** | | **Surgery inpatients in high-level hospitals (Ten thousand)** | | **Proportion of surgery outpatients in primary hospitals** | |
| --- | --- | --- | --- | --- | --- | --- | --- |
|  |  | **Before**  **COVID-19** | **COVID-19 era (Percentage change, %)** | **Before COVID-19** | **COVID-19 era (Percentage change, %)** | **Before COVID-19** | **COVID-19 era (Percentage change, %)** |
| Phase 1 | 1 | 11.05 | 9.92 (-10.24) | 13.11 | 13.30 (1.46) | 3.89% | 5.07% (1.21%) |
|  | 2 | 7.36 | 3.52 (-52.15) | 9.65 | 3.36 (-65.23) | 4.16% | 4.65% (0.45%) |
|  | 3 | 8.98 | 4.69 (-47.81) | 14.92 | 4.21 (-71.78) | 4.46% | 5.44% (0.98%) |
|  | 4 | 8.97 | 5.29 (-40.97) | 13.94 | 7.79 (-44.11) | 4.47% | 7.58% (3.12%) |
|  | **Subtotal** | **9.09** | **5.85 (-35.59)** | **12.90** | **7.16 (-44.49)** | **4.25%** | **5.68% (1.44%)** |
| Phase 2 | 5 | 9.15 | 7.02 (-23.32) | 14.67 | 9.74 (-33.63) | 4.58% | 8.03% (3.44%) |
|  | 6 | 8.81 | 7.56 (-14.18) | 13.55 | 11.68 (-13.79) | 5.15% | 6.66% (1.45%) |
|  | 7 | 8.95 | 7.18 (-19.77) | 14.55 | 10.75 (-26.09) | 5.41% | 6.32% (0.67%) |
|  | 8 | 8.92 | 8.2 (-8.11) | 14.29 | 13.52 (-5.38) | 5.57% | 6.76% (0.96%) |
|  | **Subtotal** | **8.96** | **7.49 (-16.4)** | **14.27** | **11.42 (-19.92)** | **5.18%** | **6.94% (1.63%)** |
| Phase 3 | 9 | 8.70 | 8.86 (1.83) | 14.23 | 15.56 (9.35) | 5.42% | 6.13% (0.45%) |
|  | 10 | 8.64 | 8.83 (2.17) | 12.12 | 12.6 (3.92) | 5.43% | 6.54% (0.81%) |
|  | 11 | 8.95 | 8.63 (-3.53) | 14.94 | 15.65 (4.81) | 5.44% | 6.63% (0.92%) |
|  | 12 | 11.14 | 8.46 (-24.09) | 15.11 | 16.54 (9.49) | 5.61% | 6.46% (0.54%) |
|  | **Subtotal** | **9.36** | **8.69 (-7.09)** | **14.10** | **15.09 (7.02)** | **5.47%** | **6.44% (0.68%)** |
| 2021  Phase 4&5 | 1 | 11.05 | 7.29 (-34.04) | 13.11 | 12.96 (-1.13) | 3.89% | 5.82% (1.96%) |
|  | 2 | 7.36 | 6.19 (-15.81) | 9.65 | 9.18 (-4.92) | 4.16% | 6.15% (1.96%) |
|  | 3 | 8.98 | 8.31 (-7.42) | 14.92 | 16.93 (13.46) | 4.46% | 6.63% (2.18%) |
|  | 4 | 8.97 | 9.08 (1.3) | 13.94 | 18.22 (30.78) | 4.47% | 6.64% (2.18%) |
|  | 5 | 9.15 | 9.86 (7.76) | 14.67 | 16.12 (9.89) | 4.58% | 6.80% (2.21%) |
|  | 6 | 8.81 | 10.09(14.52) | 13.55 | 17.45 (28.78) | 4.58% | 6.71% (1.56%) |
|  | 7 | 8.95 | 9.8(9.51) | 14.55 | 18.07 (24.17) | 5.15% | 6.55% (1.14%) |
|  | 8 | 8.92 | 9.05(1.44) | 14.29 | 16.92 (18.37) | 5.41% | 7.83% (2.26%) |
|  | 9 | 8.70 | 9.02(3.66) | 14.23 | 16.65 (17.03) | 5.57% | 6.65% (1.23%) |
|  | 10 | 8.64 | 9.85(14.06) | 12.12 | 14.24 (17.49) | 5.42% | 6.86% (1.43%) |
|  | **Subtotal** | **8.95** | **8.86(-1.08)** | **13.50** | **15.67 (16.08)** | **4.58%** | **6.66% (1.81%)** |

## Supplementary Table 3 The number of outpatients, proportion of outpatients in primary hospitals and non-local patients in primary or high-level hospitals before- and during COVID-19

| **Phase** | **Month** | **The proportion of outpatients in primary hospitals** | | **Outpatients in primary hospitals (Hundred thousand)** | | **Outpatients in high-level hospitals (Hundred thousand)** | | **Non-local outpatients in high-level hospitals (Hundred thousand)** | | **Non-local inpatients in high-level hospitals (Ten thousand)** | |
| --- | --- | --- | --- | --- | --- | --- | --- | --- | --- | --- | --- |
|  |  | **Before COVID-19** | **After COVID-19 (Percentage change, %)** | **Before COVID-19** | **After COVID-19 (Percentage change, %)** | **Before COVID-19** | **After COVID-19 (Percentage change, %)** | **Before COVID-19** | **After COVID-19 (Percentage change, %)** | **Before COVID-19** | **After COVID-19 (Percentage change, %)** |
| Phase 1 | 1 | 18.87% | 21.71% (2.59%) | 17.88 | 17.36 (-2.92%) | 104.98 | 86.7 (-17.41%) | 19.34 | 14.85 (-23.19%) | 11.04 | 10.14 (-8.19%) |
|  | 2 | 19.53% | 36.71% (17.35%) | 15.66 | 13.5 (-13.78%) | 87.04 | 30.26 (-65.24%) | 15.36 | 3.58 (-76.69%) | 8.07 | 2.39 (-70.32%) |
|  | 3 | 19.23% | 34.74% (15.54%) | 21.13 | 15.51 (-26.6%) | 118.94 | 39.04 (-67.17%) | 22.35 | 5.43 (-75.72%) | 12.08 | 2.36 (-80.50%) |
|  | 4 | 20.31% | 29.82% (9.43%) | 20.77 | 17.91 (-13.75%) | 109.26 | 54.64 (-49.99%) | 20.11 | 7.50 (-62.72%) | 11.67 | 3.28 (-71.92%) |
|  | **Subtotal** | **19.48%** | **29.04% (9.51%)** | **18.86** | **16.07 (-14.79%)** | **105.06** | **52.66 (-49.87%)** | **19.29** | **7.84 (-59.36%)** | **10.71** | **4.54 (-57.62%)** |
| Phase 2 | 5 | 20.5% | 27.46% (6.91%) | 21.37 | 18.65 (-12.73%) | 111.43 | 66.97 (-39.9%) | 20.50 | 11.10 (-45.83%) | 11.96 | 4.84 (-59.55%) |
|  | 6 | 20.78% | 27.12% (6.32%) | 20.65 | 18.66 (-9.64%) | 105.98 | 70.29 (-33.68%) | 19.26 | 13.07 (-32.13%) | 11.47 | 6.48 (-43.48%) |
|  | 7 | 21.25% | 26.56% (5.25%) | 22.01 | 19.41 (-11.84%) | 110.72 | 73.54 (-33.58%) | 21.24 | 13.29 (-37.41%) | 12.32 | 5.55 (-54.97%) |
|  | 8 | 21.26% | 24.74% (3.44%) | 22.41 | 20.44 (-8.79%) | 112.36 | 85.91 (-23.55%) | 21.31 | 16.25 (-23.74%) | 12.28 | 7.87 (-35.92%) |
|  | **Subtotal** | **20.95%** | **26.38% (5.40%)** | **21.61** | **19.29 (-10.74%)** | **110.12** | **74.17 (-32.64%)** | **20.58** | **13.43 (-34.73%)** | **12.00** | **6.18 (-48.49%)** |
| Phase 3 | 9 | 21.41% | 24.82% (3.36%) | 22.96 | 22.89 (-0.31%) | 110.34 | 95.34 (-13.6%) | 19.13 | 17.94 (-6.22%) | 11.69 | 9.36 (-19.99%) |
|  | 10 | 22.43% | 26.14% (3.65%) | 22.48 | 22.16 (-1.41%) | 103.58 | 87.74 (-15.29%) | 18.10 | 17.01 (-6.02%) | 10.08 | 7.94 (-21.29%) |
|  | 11 | 21.97% | 24.87% (2.88%) | 24.89 | 23.75 (-4.56%) | 116.65 | 98.28 (-15.75%) | 20.10 | 18.74 (-6.78%) | 11.99 | 9.68 (-19.31%) |
|  | 12 | 22.95% | 25.95% (2.97%) | 29.74 | 28.72 (-3.42%) | 130.84 | 109.24 (-16.51%) | 20.66 | 19.70 (-4.66%) | 12.08 | 10.42 (-13.71%) |
|  | **Subtotal** | **22.19%** | **25.45% (3.19%)** | **25.02** | **24.38 (-2.54%)** | **115.35** | **97.65 (-15.35%)** | **19.50** | **18.35 (-5.90%)** | **11.46** | **9.35 (-18.44%)** |
| 2021  Phase 4&5 | 1 | 18.87% | 21.71% (2.59%) | 17.88 | 16.06 (-10.22%) | 104.98 | 82.37 (-21.54%) | 19.34 | 17.96 (-7.14%) | 11.04 | 7.15 (-35.26%) |
|  | 2 | 19.53% | 22.14% (2.78%) | 15.66 | 14.88 (-4.99%) | 87.04 | 72.72 (-16.46%) | 15.36 | 14.83 (-3.42%) | 8.07 | 4.98 (-38.3%) |
|  | 3 | 19.23% | 21.72% (2.52%) | 21.13 | 21.85 (3.40%) | 118.94 | 107.83 (-9.34%) | 22.35 | 21.88 (-2.1%) | 12.08 | 9.84 (-18.57%) |
|  | 4 | 20.31% | 22.51% (2.12%) | 20.77 | 22.24 (7.07%) | 109.26 | 105.53 (-3.42%) | 20.11 | 21.07 (4.80%) | 11.67 | 11.52 (-1.27%) |
|  | 5 | 20.5% | 22.72% (2.18%) | 21.37 | 21.51 (0.64%) | 111.43 | 102.24 (-8.25%) | 20.50 | 20.33 (-0.83%) | 11.96 | 10.55 (-11.78%) |
|  | 6 | 20.78% | 20.79% (1.01%) | 20.65 | 21.47 (3.97%) | 105.98 | 107.69 (1.61%) | 19.26 | 21.7 (12.65%) | 11.47 | 11.44 (-0.27%) |
|  | 7 | 21.25% | 22.38% (1.13%) | 22.01 | 22.18 (0.75%) | 110.72 | 109.19 (-1.38%) | 21.24 | 23.57 (10.95%) | 12.32 | 12.13 (-1.48%) |
|  | 8 | 21.26% | 22.26% (1.00%) | 22.41 | 22.97 (2.47%) | 112.36 | 111.9 (-0.41%) | 21.31 | 23.76 (11.51%) | 12.28 | 10.51 (-14.39%) |
|  | 9 | 21.41% | 22.41% (0.99%) | 22.96 | 23.02 (0.25%) | 110.34 | 110.16 (-0.16%) | 19.13 | 22.51 (17.67%) | 11.69 | 10.94 (-6.43%) |
|  | 10 | 22.43% | 23.31% (0.88%) | 22.48 | 22.7 (1.01%) | 103.58 | 106.25 (2.58%) | 18.10 | 22.67 (25.29%) | 10.08 | 9.39 (-6.9%) |
|  | **Subtotal** | 20.56% | **22.29%** (1.74%) | **20.73** | 20.89 (0.74%) | 107.46 | 101.59 (-5.47%) | 19.67 | 21.03 (6.91%) | 11.27 | 9.84 (-12.61%) |
